# Endogenous 2μ Plasmid Editing for Pathway Engineering in *Saccharomyces cerevisiae*

**DOI:** 10.3389/fmicb.2021.631462

**Published:** 2021-02-16

**Authors:** Bo-Xuan Zeng, Ming-Dong Yao, Wen-Hai Xiao, Yun-Zi Luo, Ying Wang, Ying-Jin Yuan

**Affiliations:** ^1^Frontier Science Center for Synthetic Biology and Key Laboratory of Systems Bioengineering (Ministry of Education), School of Chemical Engineering and Technology, Tianjin University, Tianjin, China; ^2^Collaborative Innovation Center of Chemical Science and Engineering (Tianjin), Tianjin University, Tianjin, China; ^3^Department of Gastroenterology, State Key Laboratory of Biotherapy, West China Hospital, Sichuan University, Chengdu, China

**Keywords:** 2μ plasmid, CRISPR, plasmid copy number, dihydroartemisinic acid, *Saccharomyces cerevisiae*

## Abstract

In *Saccharomyces cerevisiae*, conventional 2μ-plasmid based plasmid (pC2μ, such as pRS425) have been widely adopted in pathway engineering for multi-copy overexpression of key genes. However, the loss of partition and copy number control elements of yeast endogenous 2μ plasmid (pE2μ) brings the issues concerning plasmid stability and copy number of pC2μ, especially in long-term fermentation. In this study, we developed a method based on CRISPR/Cas9 to edit pE2μ and built the pE2μ multi-copy system by insertion of the target DNA element and elimination of the original pE2μ plasmid. The resulting plasmid pE2μRAF1 and pE2μREP2 demonstrated higher copy number and slower loss rate than a pC2μ control plasmid pRS425RK, when carrying the same target gene. Then, moving the essential gene *TPI1* (encoding triose phosphate isomerase) from chromosome to pE2μRAF1 could increase the plasmid viability to nearly 100% and further increase the plasmid copy number by 73.95%. The expression using pE2μ multi-copy system demonstrated much smaller cell-to-cell variation comparing with pC2μ multi-copy system. With auxotrophic complementation of *TPI1*, the resulting plasmid pE2μRT could undergo cultivation of 90 generations under non-selective conditions without loss. Applying pE2μ multi-copy system for dihydroartemisinic acid (DHAA) biosynthesis, the production of DHAA was increased to 620.9 mg/L at shake-flask level in non-selective rich medium. This titer was 4.73-fold of the strain constructed based on pC2μ due to the more stable pE2μ plasmid system and with higher plasmid copy number. This study provides an improved expression system in yeast, and set a promising platform to construct biosynthesis pathway for valuable products.

## Introduction

**FIGURE 1:**
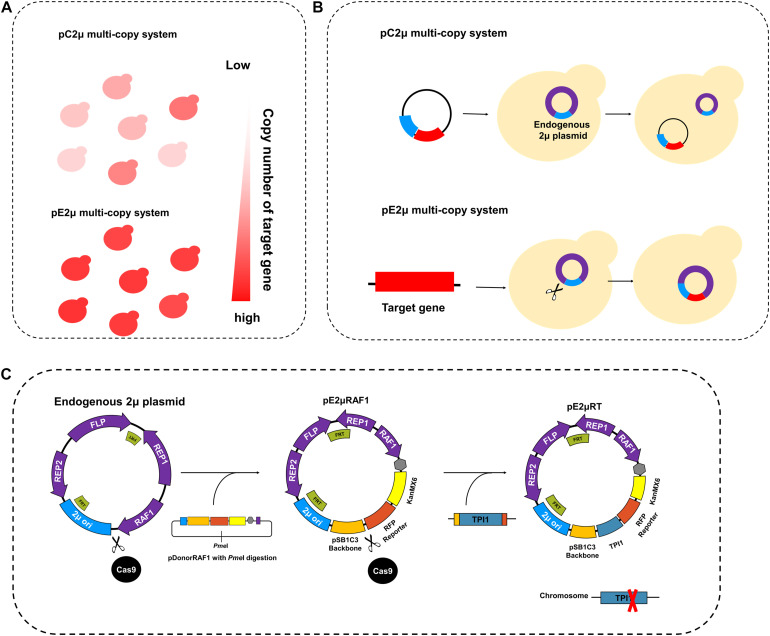
Design and construction of pE2μ multi-copy system. **(A)** The comparation of the copy number of the inserted target gene using pC2μ (conventional 2μ-based plasmid) derivate plasmid and pE2μ (endogenous 2μ plasmid) derivate plasmid. **(B)** The process of pC2μ multi-copy system and pE2μ multi-copy system. The 2μ ori (original of replication of wild type endogenous plasmid) is shown in blue, other parts of endogenous plasmid are shown in purple, the Target gene of interest is shown in red. The scissors represent the CRISPR/Cas9 tool to edit the plasmid. **(C)** The construction and optimization of the pE2μ derivate plasmid.

For the high transformation efficiency and easy manipulation, plasmids have been developed as important tools and were widely applied in many kinds of organisms (Mignon et al., 2015; Lian et al., 2016). The multi-copy plasmids are always used to overexpress genes of interest (Kang et al., 2018). In *Saccharomyces cerevisiae*, conventional 2μ-based plasmid (pC2μ) such as pYES2, pRS426, and pESC were widely used multi-copy plasmids for production of recombinant proteins as well as construction of metabolic pathway. For example, overexpression of Syn_ALD (aldehyde dehydrogenase of *Synechocystis sp.* PCC6803) and CCD2 (carotenoid cleavage dioxygenase of *Crocus*) by pRS426 increased crocetin production by about 1-fold (Chai et al., 2017). However, like all kinds of multi-copy plasmids, the plasmid viability in cells always depends on the selectivity pressure generated by the medium (with antibiotics or auxotroph medium) (Karim et al., 2013; Lian et al., 2016). In condition without selective pressure, pC2μ hardly keeps its high copy number and are difficult to be maintained in the host cell (Figure 1A). The loss frequency could reach to about 5% per generation (Gnügge and Rudolf, 2017), and nearly 50–60% of the cells lose their plasmid after about 24 h cultivation in non-selective medium (Christianson et al., 1992). For strain harboring pC2μ with auxotroph marker, the rich medium such as YPD is not suitable for long-term fermentation because of the loss of the plasmid, although the strain grows faster in these kinds of media; And for that with antibiotic marker, the addition of expensive drugs is inevitable and it is not economic for fermentation of industrial scale.

Plasmid pC2μ was derived from the yeast endogenous 2μ plasmid (pE2μ) which presents in most wild-type and laboratory *S. cerevisiae* strain (Gnügge and Rudolf, 2017). The pE2μ is a selfish episomal circular DNA element in *S. cerevisiae* (Rizvi et al., 2017a). It contains a special replication origin (2μ ori), two FRT sites (*FLP1* recognized target site), and sequences encoding four known genes: *REP1*, *REP2*, *RAF1*, and *FLP1* (Gnügge and Rudolf, 2017; McQuaid et al., 2017; Rizvi et al., 2017a; see Supplementary Figure 1). *FLP1* and both FRT sites are essential for the amplification of pE2μ. This amplification system is based on the *FLP1*-mediated recombination and follows the special Futcher’s model (Futcher, 1986; Rizvi et al., 2017a), thus each plasmid could reproduce more than one copy in one cell cycle to restore the steady-state for plasmid copy number (PCN) under missegregation and consequent perturbations (Rizvi et al., 2017a). Whereas, pC2μ plasmid contains only one FRT site. So that pC2μ cannot be amplified as Futcher’s model itself, and the amplification of pC2μ is not efficient as pE2μ. Therefore, the PCN of pC2μ cannot compete with native pE2μ, even though weaken the expression of the selective marker by truncated or weaker promoter could increase the average plasmid copy number of pC2μ by eliminating the cells with less plasmid (Karim et al., 2013).

*REP1* and *REP2* are essential for the partition system of pE2μ. *REP1* and *REP2* form the *REP1-REP2* complex which binds to the *cis*-acting locus (STB locus) (see Supplementary Figure 1) located within the 2μ ori to ensure equal segregation of plasmids from mother to daughter cells (Gnügge and Rudolf, 2017; Rizvi et al., 2017a); *RAF1* is responsible for the regulation of partition and amplification system to keep the plasmid copy number of pE2μ and minimize the cell-to-cell variations (McQuaid et al., 2017; Rizvi et al., 2017b). Based on the tightly controlled partition system and amplification system described above, pE2μ could be segregated equally and the copy number could be restored in some missegregation events, leading to very low loss frequency (<0.01%) (Gnügge and Rudolf, 2017). The pC2μ only contains 2μ ori and does not contain *REP1* and *REP2*, so the maintenance of pC2μ has to depend on pE2μ (Figure 1B). Rising from segregation instability, the loss frequency of pC2μ is about 50% per generation in strains without pE2μ (Gnügge and Rudolf, 2017). Therefore, to achieve stable and high-level expression of the targeted genes, we intended to employ pE2μ rather than pC2μ as an expression vector. Yukie Misumi once constructed the plasmid YHp (Misumi et al., 2019) based on the structure of pE2μ and applied in the *S. cerevisiae* strain without endogenous 2μ plasmid. However, because of the similar structure, YHp is not compatible with wild type pE2μ and was not suitable for application in commonly used laboratory *S. cerevisiae* strain which harboring wild type pE2μ.

In our work, we developed a novel multi-copy plasmid system which based on editing pE2μ by CRISPR/Cas9 genome engineering tools (Figures 1B,C). The laboratory strain CEN.PK2-1C was chosen as the host. It contains the natural wild type pE2μ plasmid. By using this pE2μ multi-copy plasmid system, the target gene could be overexpressed more stable and at higher level than using the commonly used pC2μ multi-copy plasmid system (Figure 1A). Although the edited pE2μ was still lost during long-term cultivation in non-selective medium, the plasmid viability was much higher than that of pC2μ. To further reduce the loss frequency of pE2μ, auxotrophy complementation strategy was applied by introducing the essential gene *TPI1* (encoding triose phosphate isomerase) into pE2μ by CRISPR/Cas9 system as well as knocking out the *TPI1* in the chromosome (Figure 1C). As a result, pE2μ plasmid (pE2μRT) could be maintained in cells for long-term cultivation. And its PCN was also increased. Although p425RT (pC2μ derived plasmid carrying *TPI1*) in strain without chromosome *TPI1* could also be maintained in cell after long-term cultivation in YPD, the average level and stability of p425RT could not compared with that of pE2μRT. At last, we applied the method for optimization of the metabolic pathway of dihydroartemisinic acid (DHAA) production in non-selective medium. The production was successfully increased to 620.9 mg/L which was 4.73-fold higher than the strain using pC2μ to overexpress the gene of the same biosynthetic pathway.

## Materials and Methods

### Strains and Medium

*Escherichia coli* DH5α was used for construction of all the plasmids and was cultured at 37°C in Luria-Bertani (LB) medium (1% tryptone, 0.5% yeast extract, and 1% NaCl) with 100 μg/ml ampicillin or 34 μg/ml chloramphenicol if necessary. All engineered yeast strains were derived from *S. cerevisiae* CEN.PK2-1C (Entian and Ktter, 2007) obtained from EUROSCARF (Frankfurt, Germany). The yeast strains were cultured at 30°C in YPD medium (2% peptone, 1% yeast extract, and 2% glucose) or in synthetic complete (SC) drop-out medium. G418 was added to medium in final concentration of 200 μg/ml if necessary. The strains for DHAA production was derivative from Sc027 (Zeng et al., 2020). All *S. cerevisiae* strains used in this study were listed in Table 1.

**TABLE 1:** Yeast strains used in this study.

**Yeast Strains**	**Description**	**Source**
CEN.PK2-1C	*MAT a; ura3-52, trp1-289, leu2-3,112, his3Δ1, MAL2-8C, SUC2*	Invitrogen
Sc382	*CEN.PK2-1C* derivative*; Δ2μ:: pE2μRAF1*	This study
Sc534	*CEN.PK2-1C* derivative*; pRS425RK*	This study
Sc438	*CEN.PK2-1C* derivative*; Δ2μ::pE2μREP2*	This study
Sc591	*Sc382 derivative; ΔE2μRAF1::pE2μRT*	This study
Sc594	*Sc591* derivative*; ΔTPI1(chromosome)::leu2*	This study
Sc530	*CEN.PK2-1C derivative; p425RT; ΔTPI1(chromosome)::his3*	
Sc027	CEN.PK2-1C derivative*; leu2-3,112::KanMX6_P_GAL7_-CYB5_T_ERG19_(RC)-ERG19(RC)-P_*GAL1*_(RC)_P_GAL10_-ERG8-T_ERG8_; his3Δ1::HIS3_P_GAL7_-ALDH1-T_*TDH*1__*T*_*ERG12*_(RC)-ERG12(RC)-P_GAL1_(RC)_P_GAL10_-ERG10-T_ERG10_*; *ade1Δ::T_HMG1_(RC)-tHMG1(RC)-P_GAL1_(RC)_P_GAL10_-IDI1-T_IDI1__ADE1; ura3-52::T_HMG1_(RC)-tHMG1(RC)-P_GAL1_(RC)_P_GAL10_-ERG13-T_ERG13_; trp1-289::T_HMG1_(RC)-tHMG1(RC)-P_GAL1_(RC)_P_GAL10_-ERG20-T_ERG20__TRP1; gal1/10/7Δ::natA_P_GAL3_-CPR1-T_CYC1_*;	Zeng et al., 2020
Sc341	*Sc027 derivative; Δgal80::P_CYC1_-GAL4-T_*GAL4*__*P*_*GAL7*_-ADH1-T_TDH1_; ΔKanMX6::P_TDH1_-HEM1-T_*HEM1*__*P*_*PGK1*_-CTT1-T_CTT1__hphA*	This study
Sc366	*Sc341 derivative; p425DCA*	This study
Sc343	*Sc341 derivative; Δ2μ::pE2μDCA*	This study
Sc582	*Sc343 derivative; ΔpE2μDCA::pE2μDCAT*	This study
Sc584	*Sc582 derivative; ΔTPI1::leu2*	This study

### Construction of pE2μ Derivate Plasmid

All the plasmids constructed in this study were listed in Table 2. The primer used in this study were listed in Supplementary Table 1.

**TABLE 2:** Plasmids used in this study.

**Plasmid**	**Description**	**Source**
pNA0306	*pRS415_P_TEF1–_Cas9-T_CYC1_*	Xie et al., 2018
pNA0304	*pRS426-P_SNR52_-gRNA-T_SUP4_*	Xie et al., 2018
pCasRAF1	*pRS416_P_TEF1_-Cas9-T_*CYC1*__*P*_*SNR52*_-gRNA.2μ.RAF1-T_SUP4_-T_*CYC1*_*	This study
pCasREP2	*pRS416_P_TEF1_-Cas9-T_*CYC1*__*P*_*SNR52*_-gRNA.2μ.REP2-T_SUP4_-T_CYC1_*	This study
pCasE2μ	*pRS416_P_TEF1_-Cas9-T_*CYC1*__*P*_*SNR52*_-gRNA.E2μ-T_SUP4_-T_CYC1_*	This study
pDonorRAF1	*pSB1C3_P_TDH3_-RFP-T_ADH1__KanMX6(RC)_T_PGI1_(RC)_homoRAF1(RC)_T_RAF1_ (RC)*	This study
pDonorREP2	*pSB1C3_P_TDH3_-RFP-T_ADH1__KanMX6(RC)_T_CPS__1_ (RC)_homoREP2(RC)_T_REP2_ (RC)*	This study
pE2μRAF1	*pSB1C3_P_TDH3_-RFP-T_ADH1__KanMX6(RC)_T_PGI1_(RC)-RAF1(RC)-P_RAF1_(RC)_rep1_FRT(RC)_flp1(RC)_rep2_2μ ori*	This study
pE2μREP2	*pSB1C3_P_*TDH3*_-RFP-T_ADH1__KanMX6(RC)_T_PGI1_(RC)-RAF1(RC)-P_RAF1_(RC)_rep1_FRT(RC)_flp1(RC)_rep2_2μ ori*	This study
pRS425RK	*pRS425_ P_TDH3_-RFP-T_ADH1_(RC)_KanMX6(RC)*	This study
pE2μRT	*pSB1C3_P_TPI1_(RC)-TPI1(RC)-T_TPI1_(RC)_P_TDH3_-RFP-T_ADH1__G418R(RC)_T_PGI1_(RC)-RAF1(RC)-P_RAF1_(RC)_rep1_FRT(RC)_flp(RC)_rep2_2μ ori*	This study
p425RT	*pRS425_ P_TPI1_(RC)-TPI1(RC)-T_TPI1_(RC)_P_*TDH3*_-RFP-T_ADH1_*	This study
p425DCA	pRS425_T_CYC1_(RC)-DBR2(RC)-P_GAL10_(RC)-_P_*GAL1*_-ADS-T_*PGK1*__*P*_*GAL7*_-CYP71AV1*-T_ADH1_	This study
pDonorDCA	pSB1C3_ T_CYC1_(RC)-DBR2(RC)-P_GAL10_(RC)-_P_GAL1_-ADS-T_*PGK1*__*P*_*GAL7*_-CYP71AV1*-T_ADH1__KanMX6(RC)_T_*PGI1*_(RC)_homoRAF1(RC)_PmeI_T_*RAF1*_ (RC)	This study
pE2μDCA	pSB1C3_ T_CYC1_(RC)-DBR2(RC)-P_GAL10_(RC)-_P_GAL1_-ADS-T_*PGK1*__*P*_*GAL7*_-CYP71AV1*-T_ADH1__KanMX6(RC)_T_PGI1_(RC)_ RAF1(RC)-P_RAF1_(RC)_rep1_FRT(RC)_flp1(RC)_rep2_2μ ori	This study
pE2μDCAT	pSB1C3_ P_TPI1_(RC)-TPI1(RC)-T_TPI1_(RC)_T_CYC1_(RC)-DBR2(RC)-P_GAL10_(RC)-_P_GAL1_-ADS-T_*PGK1*__*P*_*GAL7*_-CYP71AV1*-T_ADH1__KanMX6(RC)_T_PGI1_(RC)_ RAF1(RC)-P_RAF1_(RC)_rep1_FRT(RC)_flp1(RC)_rep2_2μ ori	This study
stALG2	*pRS425_ALG9(partial)_ P_TDH3_-RFP-T_ADH1__KanMX6(RC)*	This study
pSB1C3	*Backbone for plasmid construction, CmR*	Zeng et al., 2020
pRS416	*Backbone for plasmid construction. Amp, Ura3*	GenBank: U03450.1

The DNA fragment followed by “(RC)” represented the orientation of the DNA fragment was reversed. CYP71AV1*: all CYP71AV1 used in this study was optimized by replacing the N-terminal membrane anchoring sequence (15-residue peptide) with the more hydrophilic 8-residue peptide from bovine (8RP) (Chen et al., 2017).

Three CRISPR/Cas9 plasmid pCasRAF1, pCasREP2, and pCasE2μ were constructed based on pRS416 (Supplementary Figure 2). Took the construction of pCasRAF1 for example: P_*SNR52*_(RAF1) was amplified from pNA0304 by PCR using primer 18Q3-pSNR52-F and 18Q0b-pSNR52-R; Cassette *gRNA.2μ.RAF1-T_*SUP4*_-T_*CYC1*_* was amplified from pNA0304 by PCR using primer 18Q0b-gRNA-F and 18Q3-cyc1t-R; Two DNA fragment were assembled together to constructed cassette *P_SNR52_-gRNA.2μ.RAF1-T_SUP4_-T_CYC1_* by OE-PCR (overlap extension PCR) using primer 18Q3-cyc1t-R and 18Q3-pSNR52-F. Cassette *P_TEF1_-cas9-T_CYC1_* was amplified by PCR from pNA0306 using primer 18Q3-cas9-F and 18Q3-cas9-R. Cassette *P_SNR52_-gRNA.2μ.RAF1-T_SUP4_-T_CYC1_* was digested by *Sph*I and *Not*I; Cassette *P_TEF1_-Cas9-T_CYC1_* was digested by *Kpn*I and *Sph*I; pRS416 was digested by *Kpn*I and *Not*I. Three digested DNA fragments were ligated together by T4 ligase to construct pCasRAF1. pCasREP2 and pCasE2μ were constructed in similar way.

Plasmids pDonorRAF1, pDonorREP2, pDonorDCA were donor plasmid. Took the construction of pDonorRAF1 for example: T_*GPI1*_, homoRAF1, T_*RAF1*_ were amplified by PCR from the genome of CEN.PK2-1C using primers 18Q2a-pgi1t-R/18Q2a-pgi1t-F, 18Q2a-homodown-F/18Q2a-homodown-R, 18Q2a-homoup-F/18Q2a-homoup-R. The DNA fragment were assembled to construct *T_PGI1__homoRAF1_PmeI_T_RAF1_* by OE-PCR and amplified using primer 18Q2a-pgi1t-F/18Q2a-homoup-R. Cassette *P_TDH3_-RFP-T_ADH1_* was amplified by PCR using primer 18Q4-pTDH3-F/18Q4-adh1t-R. Cassette *KanMX6* was amplified by PCR using primer 18Q4a-kanMX6-F/18Q4a-kanMX6-R. Cassette *T_PGI1__homoRAF1_PmeI_T_RAF1_* was digested by PstI and XbaI; *P_TDH3_-RFP-T_ADH1_* was digested by *EcoR*I and *Xho*I; *KanMX6* was digested by *Xho*I and *Xba*I; plasmid pSB1C3 was digested by *Pst*I and *EcoR*I. All 4 digested fragment were ligated together by T4 ligase to construct pDonorRAF1. Donor plasmid pDonorREP2 and pDonorDCA was constructed in similar way.

For construction of the *S. cerevisiae* strain Sc382, donor plasmid pDonorRAF1 was linearized by *Pme*I and was co-transformed to CEN.PK2-1C with CRISPR/Cas9 plasmid pCasRAF1 to insert the whole donor DNA into endogenous 2μ plasmid. The SC-Ura plate with 200 mg/L G418 was used for selection of the correct transformants. After keeping the plate at 30°C for about 72 h, all the transformants were visible. The single colonies were picked up and transferred to SC-Ura plate with 200 mg/L G418 and incubated at 30°C for 24 h. Then, the colonies were identified by colony PCR. For strain Sc382, the primer pairs 2μori-test-F/BioBrick-R and 18Q-test-5/18Q-test-15 were used for verification of the insertion of the donor DNA. The primer pair 2μori-test-F/18Q-test-5 was used for verification of the elimination of all wild type endogenous 2μ plasmid. The result was used for characterization of the efficiency of the method of editing endogenous 2μ plasmid by CRISPR/Cas9 system. After verification, the correct colonies were steaked on 5-FOA (5-Fluoroorotic acid) plate with 200 mg/L G418 to lose the pCasRAF1 plasmid.

The process of construction of strain Sc438 and Sc343 were similar to that of strain Sc382. Donor plasmid pDonorREP2 and pCasREP2 were used for co-transformation into CEN.PK2-1C to construct strain Sc438 (harboring plasmid pE2μREP2); Donor plasmid pDonorDCA and pCasRAF1 were used for co-transformation into CEN.PK2-1C to construct strain Sc343 (harboring plasmid pE2μDCA).

To introduce *TPI1* to pE2μ, DNA fragment tpi1-1 was amplified from genome of CEN.PK2-1C by PCR using primer 20QRcT-R4/20QRcT-F4, tpi1-2 was amplified from tpi1-1 by PCR using primer 20QRcT-R5/20QRcT-F5, tpi1-3 was amplified from tpi1-2 by PCR using primer 20QRcT-R6/20QRcT-F6. The DNA fragment tpi1-3 was co-transformed to strain Sc382 (containing pE2μRAF1) and Sc343 (containing pE2μDCA) with CRISRP/Cas9 plasmid pCasE2μ to insert *TPI1* into pE2μRAF1 or pE2μDCA to construct strain Sc591 and Sc582.

To delete the *TPI1* of chromosome, leu2-1 was amplified from pRS425 by PCR using primer dTPI1-leu2-F1/dTPI1-leu2-R1; Then, leu2-2 was amplified from leu2-1 by PCR using primer dTPI1-leu2-F2/dTPI1-leu2-R2; and leu2-3 was amplified from leu2-2 by PCR using primer dTPI1-leu2-F3/dTPI1-leu2-R3. DNA fragment leu2-3 was introduced to Sc591 and Sc582 for deletion of TPI1 to construct strain Sc594 and Sc584. For construction of strain Sc530, p425RT was introduced into CEN.PK2-1C and then chromosome *TPI1* was deleted by *his3.*

### Plasmid Stability Assay

To characterize the property of plasmid pE2μ and pC2μ, strain Sc382, Sc438, Sc534, Sc530, and Sc594 were tested in non-selective YPD medium. The single colony from selective YPD+G418 medium plate was inoculated to 3 ml YPD+G418 medium (for Sc530, the medium was SC-Leu). The saturated culture was re-inoculated to 3 ml YPD+G418 medium (for Sc530, the medium was SC-Leu) at an optical density (OD_600_) 0.05 and grown for 10 h at 30°C. The culture was at 0 generation and was prepared for characterization. Then the culture was re-inoculated to non-selective YPD medium at OD_600_ = 0.05 and grown for 10 h at 30°C again to obtain the culture at 5 generation. Then serial sub-cultures were conducted every 5 generation until the 30th generation. The cultures at 5, 10, 20, and 30 generation were prepared for characterization.

For measurement of the fluorescence of RFP (Red fluorescence protein), plate reader (SpectraMAX M2, Molecular Devices) was used with a 587 nm excitation filter and a 610 nm emission filter. The fluorescence of RFP at single cell level was measured by flow cytometer (NovoCyte D2040R). The culture of each generation was diluted and plated on YPD medium and YPD+G418 medium (for Sc530, the selective medium was SC-Leu) to calculate the viability of the plasmid by counting the colony number of each plate.

### Determination of Plasmid Copy Number (PCN)

The plasmid copy number (PCN) was measured by quantitative PCR using the total DNA extracted from the strain. The process of total DNA extraction: the cells was harvest at mid-log phase, and were treated with lysis buffer (20 mM phosphate buffer at pH = 7.2, 1.2 M sorbitol, 15 U zymolyase) at 37°C for 20 min; The total DNA was extracted by boiling the sample for 15 min, −80°C for 15 min and then boiling again for 15 min (Lian et al., 2016). The suspension was diluted 10-fold before qPCR analyze. The absolute quantitative method referred to Lee et al. (2006). The plasmid stALG2 (contain ALG9, RFP, KanMX6) and p425DCA (containing ADS) was used for construction of the standard curve. TransStart^®^ Top Green qPCR SuperMix (purchased from TransGen Biotech Co., Ltd) were used for qPCR analyze on Real time fluorescent quantitative PCR (Molarray MA-6000). The primers used for qPCR were listed in Supplementary Table 1.

### Fermentation and Measurement of the DHAA

The medium used for fermentation was YPD medium and FM medium (as our previous work (Zeng et al., 2020)). The FM medium was composed of 8 g/L KH_2_PO_4_, 15 g/L (NH4)_2_SO_4_, 6.2 g/L MgSO_4_⋅7H_2_O, 40 g/L glucose, 12 ml/L vitamin solution, and 10 ml/L trace metal solution and 10 ml/L Amino acid solution. Vitamin solution included 0.05 g/L biotin, 1 g/L calcium pantothenate, 1 g/L nicotinic acid, 25 g/L myo-inositol, 1 g/L thiamine HCl, 1 g/L pyridoxal HCl, 0.2 g/L p-aminobenzoic acid, and 2 g/L adenine sulfate. Trace metal solution is composed of 5.75 g/L ZnSO_4_⋅7H_2_O, 0.32 g/L MnCl_2_⋅4H_2_O, 0.32 g/L Anhydrous CuSO_4_, 0.47 g/L CoCl_2_⋅6H_2_O, 0.48 g/L Na_2_MoO_4_⋅2H_2_O, 2.9 g/L CaCl_2_⋅2H_2_O, 2.8 g/L FeSO_4_⋅7H_2_O, and 80 ml/L EDTA solution (containing 0.5 mol/L Na_2_EDTA pH = 8.0). Amino acid solution is composed of 2 g/L methionine, 6 g/L tryptophan, 8 g/L isoleucine, 5 g/L phenylalanine, 10 g/L sodium glutamate, 20 g/L threonine, 10 g/L aspartate, 15 g/L valine, 40 g/L serine, and 2 g/L arginine. The selective medium for Sc366 was FM medium adding 50 mg/L uracil, the selective medium for Sc343 and Sc584 was FM medium adding 200 mg/L leucine, and 50 mg/L uracil and 200 mg/L G418. The non-selective medium for all strains were YPD medium. To test the productivity of the strains, the single colony from plate was inoculated to selective medium and cultured for 18–24 h at 30°C. The seeds were re-inoculated to 3 ml selective medium at OD_600_ = 0.05 and cultured for another 18–24 h at 30°C. The seed culture of each strain was transferred 250 ml flask containing 25 ml selective medium and flask containing 25 ml non-selective medium at initial OD_600_ of 0.2. The cell was grown at 30°C with shaking at 200 rpm. After 24 h, 5 ml dodecane and 20 g/L ethanol was added to each flask. The whole fermentation process continued for 120 h until harvest.

After harvest, the fermentation broth was centrifuged at 12,000 × *g* for 2 min and the dodecane phase was collected. And then 50 μL organic phase was mixed with 950 μL methanol. After filtrated with 0.22 μm Nylon 66 filter, the sample was ready for HPLC analysis. The method for HPLC analysis and the measurement of DHAA and other intermediates had been reported in previous work (Zeng et al., 2020).

### Measurement of Relative mRNA Level

During the fermentation of Sc366 and Sc343, 1 ml samples was collected at 40 h. Total RNA was extracted from the cell using Yeast RNA kit (Omega bio-tek). The Reverse transcription procedure was used TransScript^®^ First-Strand cDNA Synthesis SuperMix (purchased from TransGen Biotech Co., Ltd). The relative cDNA level of *ADS*, *CYP71AV1*, *DBR2* were measured by qPCR. *ALG9* was the reference gene, and the result was relative to that of Sc366. The primers used for qPCR were listed in Supplementary Table 1.

## Results and Discussion

### The Construction of pE2μ Multi-Copy System

In order to construct the multi-copy system, we intended to insert the target DNA into the wild type pE2μ plasmid of CEN.PK2-1C. The pDonor plasmid which harboring homologous arms of wild type pE2μ was designed to carry target DNA. After linearization, pDonor could be transformed into CEN.PK2-1C and inserted into wild type pE2μ by homologous recombination to form the recombinant plasmid. Since the incompatibility between the recombinant plasmid and the original wild type pE2μ plasmid, the CRISPR/Cas9 plasmid was designed to and enhance the recombination of pDonor with pE2μ and eliminate all the original wild type pE2μ without insertion of pDonor. The resulting strain only contained the recombinant plasmid with multi-copies (Figure 1C).

To insert the target DNA into pE2μ, the site for recombination and the editing target of CRISPR/Cas9 plasmid had to be determined. Beside the four known genes, there are several uncharacterized transcripts transcribed from wild type pE2μ plasmid (Rizvi et al., 2017a). All the elements described above covered almost the whole plasmid. To avoid the disruption of the plasmid function, only two sites can be chosen as the targets for insertion of foreign DNA fragment: One is at the downstream of the *RAF1* (Supplementary Figure 1A), another is at the end of ORF of *REP2* (Supplementary Figure 1B). The CRISPR/Cas9 plasmid pCasRAF1 and pCasREP2 were constructed for each target described above. Each of two plasmids encoded both the RNA-guided endonuclease Cas9 and the guide RNA (gRNA) of corresponding (see Supplementary Figure 2). The centromeric plasmid pRS416 was used as the backbone to construct the CRISPR/Cas9 plasmid. Although using multi-copy plasmid such as pRS426 might increase the efficiency of genome or plasmid editing and had been successfully applied for multi-genes disruption (Jakounas et al., 2015; Lian et al., 2018), the recombination between pRS426 and endogenous 2μ plasmid through FRT sites by FLP1 was not desired during the plasmid editing process.

In order to facilitate the introduction of foreign DNA and characterize the modified 2μ plasmid, we designed two donor plasmids pDonorRAF1 and pDonorREP2. The vector composed of backbone of pSB1C3, *KanMX6* for selection, RFP (Red fluorescent protein) cassette as reporter, homologous arms and a terminator (Figure 1). Different from common genome editing by CRISPR/Cas9 system, *KanMX6* is necessary selective marker for editing the wild type 2μ plasmid, because there is no essential gene on it. The terminator would help to finish the transcription of *RAF1* or *REP2* which might be influenced by inserted DNA. RFP cassette was used for characterization of the modified 2μ plasmid and two *Bsa*I restriction endonuclease sites flanked by RFP were designed for substitute RFP cassette by other gene of interest. After linearized by *Pme*I, the donor plasmid pDonorRAF1 or pDonorREP2 was co-transformed with their corresponding CRISPR/Cas9 system plasmid (pCasRAF1 or pCasREP2) into host CEN.PK2-1C. After plasmid editing and recombination, the strain Sc382 (harboring recombinant plasmid pE2μRAF1) and Sc438 (harboring recombinant plasmid pE2μREP2) were constructed (see Figure 1C).

Take the construction of Sc382 for example, we randomly picked 36 single colonies for verification by colony PCR. Among them, 22 colonies (about 61.1%) were successfully transformed by linearized DNA. And no wild type endogenous 2μ plasmid remained in these colonies. The yeast plasmids extracted from strain Sc438 and Sc382 could be successfully transformed to *E. coli* and obtain the plasmid pE2μRAF1 and pE2μREP2. The sequencing result of the 2μ related part of pE2μRAF1 and pE2μREP2 were consistent with reported endogenous 2μ plasmid of CEN.PK113-7D (GenBank: CP025735.1) (Piroon et al., 2018), and the editing process was further proved to be successful.

Although CRISPR/Cas9 system has been successfully applied in genome editing, it was the first attempt to edit the multi-copy plasmid. Comparing with the efficiency of genome editing which was almost 100% (Jakounas et al., 2015; Generoso et al., 2016), the efficiency of multi-plasmid editing was not as high as that. Multi-copy target sequences and the special amplification system of endogenous 2μ plasmid (Gnügge and Rudolf, 2017) made the work of CRISPR/Cas9 system not easy. Some strategies for optimization CRISPR/Cas9 system increasing might be helpful for increasing the efficiency of 2μ plasmid editing, such as increasing activity of cleavage by using Cas9 variants (Bao et al., 2014), changing the promoter for optimization of gRNA expression (Gao and Zhao, 2013), and facilitating gRNA-transient expression system (Easmin et al., 2019). Anyway, the method for plasmid editing applied in this work had been proved to be viable and successful to insert the target DNA element into the pE2μ to form a new recombinant multi-copy plasmid and eliminate the original pE2μ plasmid.

### Characterization of the Plasmids From pE2μ Multi-Copy System

**FIGURE 2:**
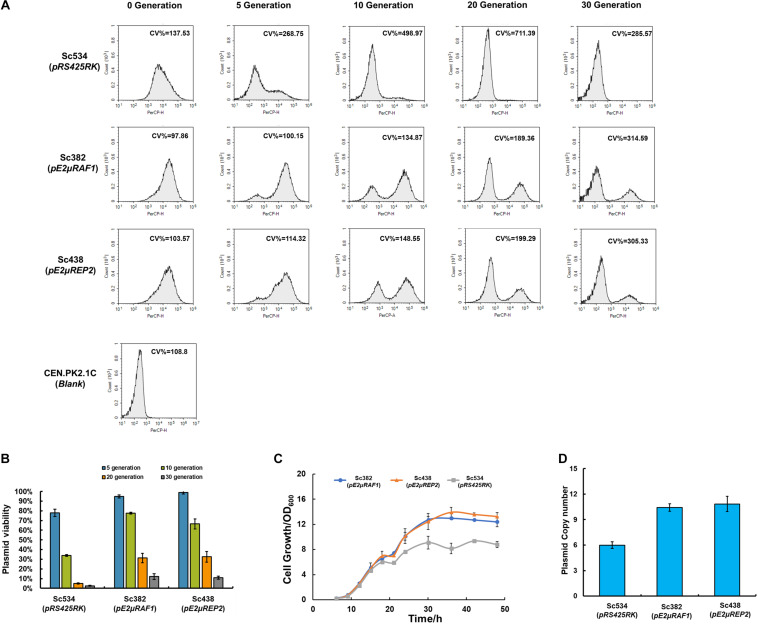
Comparing the strains harboring pC2μ derivate plasmid (pRS425RK) and stains harboring pE2μ derivate plasmid (pE2μRAF1 or pE2μREP2) when carrying RFP expression cassettes. **(A)** Fluorescence at the single-cell analyzed by flow cytometry during cultivation of 30 generations in non-selective YPD medium. Strain CEN.PK2-1C which didn’t express RFP was set as the blank. Different plasmids harbored by strain were shown in the bracket. CV %, coefficient of variance. **(B)** Comparation of PCN of different strains in selective YPD+G418 medium (at 0 generation). **(C)** The cell growth of Sc534, Sc438, and Sc382 in selective YPD+G418 medium **(D)** Comparation of plasmid stability assay for strains Sc534, Sc382, and Sc438 in non-selective YPD medium.

To characterize the property of pE2μ, we chose pC2μ plasmid pRS425RK as a control. pRS425RK was constructed based on conventional 2μ plasmid pRS425. RFP cassette and KanMX6 of pRS425RK were used for characterization. The strain Sc534 which harboring pRS425RK was constructed. We evaluated the stability of Sc382 and Sc438 compared with control strain Sc534 by plasmid stability assay. The strains were culture in YPD media without selective pressure and were transferred to fresh media every 5 generation. The fluorescence at single cell level was measured at 5, 10, 20, and 30 generation (Figure 2). Under condition with selective pressure (at 0 generation), the cells of the strains with pE2μ (Sc382 and Sc438) showed less cell-to-cell variation (smaller CV %) than that with pRS425RK (Sc534) (Figure 2A). The average fluorescence of strains Sc438 and Sc382 was also 3.42 to 3.67-fold higher than that with pRS425RK (Supplementary Figure 3). The cell growth of Sc382 and Sc438 higher than that of Sc534 after 48 h fermentation in condition with selective pressure (Figure 2C). The PCN of Sc382 (pE2μRAF1) achieved 10.4 and was about 1.67-fold higher than that of Sc534, while the average PCN of Sc438 (pE2μREP2) was 10.8 copies and also higher than that of Sc534 (Figure 2D).

After culturing in the condition without selective pressure, all the strains began to lose the plasmid. Nearly 94% cells of the Sc534 (pRS425RK) lost their plasmid after culture of 10 generation and hardly to find cells with plasmid after 20 generation. The frequency of plasmid lost is lower at strain Sc382 and Sc438. Only about 20–23% of the cells lost their plasmid before 10 generation, but 88–89% of the cells had lost their plasmid until 30 generation (Figure 2B). There was no significant difference in PCN and plasmid stability between pE2μRAF1 and pE2μREP2. Although the stability of the strain with pE2μ is higher than the strain with conventional 2μ plasmid, it is much lower than the wild type endogenous 2μ plasmid.

The PCN control system of 2μ plasmid based on *RAF1* and *REP1-REP2* complex could tightly control its copy number (McQuaid et al., 2017; Rizvi et al., 2017a). *REP1-REP2* complex is responsible for stability of plasmid and repression of FLP to control the PCN; *RAF1* could repress the formation of *REP1-REP2* to derepress the expression of *FLP1* to increase the PCN (Rizvi et al., 2017b). The pC2μ is out of this copy number control system, and difficult to keep the stability and equality even in condition with selective pressure. While, the pE2μ could facilitate the PCN control system to keep its equality of PCN, and is more stable. The insertion of foreign DNA didn’t disrupt any known genes on the 2μ plasmid, but it might influence the expression level of these genes or other unknown function transcripts to reduce the stability of 2μ plasmid. In spite of that, pE2μ is might be a better choice than pC2μ plasmid to overexpress the genes of interest by increasing its copy number. Strain Sc382 was chosen as the host for further optimization.

### Optimization of pE2μ Multi-Copy System by Auxotrophy Complementation of Essential Gene *TPI1*

**FIGURE 3:**
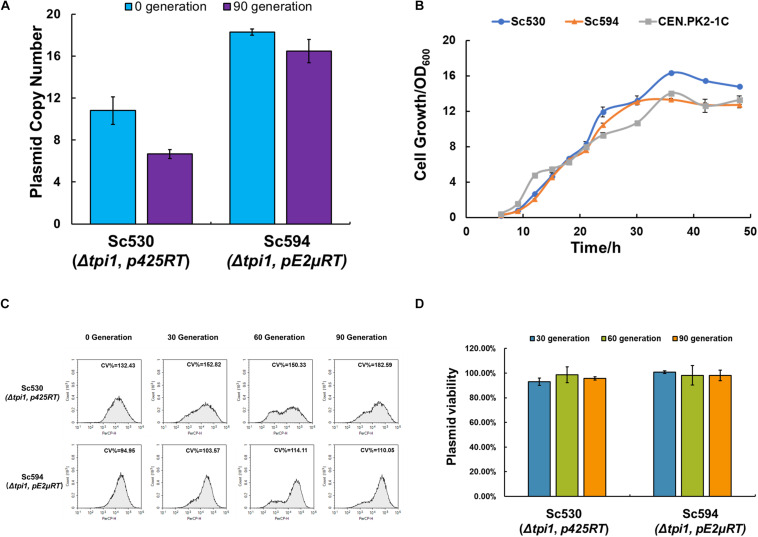
Effect of *TPI1* auxotrophic complementation on strains Sc594 and Sc530 harboring pC2μ and pE2μ derivate plasmids, respectively. **(A)** Comparation of PCN for strain Sc530 and Sc594 before (0 generation) or after (90 generation) a very long-term cultivation in non-selective YPD medium **(B)** Cell growth of strain Sc594 and Sc530 and the wild type CEN.PK2-1C in non-selective YPD medium. **(C)** Fluorescence at the single-cell analyzed by flow cytometry during cultivation of 90 generations. **(D)** The plasmid stability assay for strain Sc594 and Sc530.

The maintenance of the plasmid in the cell is very important for long-term fermentation. To eliminate the plasmid loss of pE2μ in non-selective condition, an auxotroph marker based on essential gene *TPI1* was constructed. Gene *TPI1* encodes triose phosphate isomerase which is required for growth on glucose and makes up about 2% of the soluble cellular protein (Scott and Baker, 1993). The strategy of using essential gene to substitute the one on the chromosome to construct the selective pressure-free system and optimize the plasmid system had been used in *E. coli* (Chen, 2012; Kang et al., 2018). However, fewer studies focused on the optimization of plasmid in *S. cerevisiae*. CRISPR/Cas9 plasmid pCasE2μ was constructed for insertion of *TPI1* into plasmid pE2μRAF1 of strain Sc382. The editing target of pCasE2μ was on the pSB1C3 backbone of pE2μRAF1. After insertion of *TPI1* into pE2μRAF1 (the resulting plasmid was pE2μRT) and deletion of native *TPI1* in chromosome by *his3*, the strain Sc594 was constructed (see Figure 1C). For comparation, strain Sc530 which harboring plasmid p425RT (based on pRS425 and containing *TPI1*) and the deletion of the native *TPI1* was constructed. Compared with wild type CEN.PK2-1C, both Sc594 and Sc530 showed similar cell growth for 48 h fermentation in non-selective YPD medium (see Figure 3B). The plasmid viability of both strains was evaluated by a very long-term cultivation (90 generation) without any selective pressure in YPD medium. For both strains, the viability was nearly 100% (see Figure 3D), and no plasmid-free cell was found at 90 generation, all cells plated on YPD plate were expressing RFP (see Supplementary Figure 4A). *TPI1* was the key enzyme for both glycolysis and gluconeogenesis process, cells without *TPI1* are inviable (Giaever et al., 2002). For strain Sc594, after moving the TPI1 from chromosome to plasmid, TPI1 became an auxotrophy selective marker of the plasmid. Since TPI1 is an essential gene, this auxotrophy selective marker have no requirement of the condition, and the plasmid-free cells was inviable. On the contrary, for strain Sc382 or Sc438, the selective marker of their plasmid was KanMX6, the plasmid only could be kept in condition with G418, the cells would lose the plasmid in non-selective YPD medium. Therefore, the plasmid viability of Sc594 or Sc530 was shown higher than that of Sc382 or Sc438. Optimization of the plasmid by auxotrophy complementation of essential gene *TPI1* could also increase the PCN of both pC2μ and pE2μ derivate plasmid. The PCN of pE2μRT in Sc594 achieved to about 18.3 and was 1.76-fold higher than that of pE2μRAF1 in Sc382 at 0 generation; while the PCN of p425RT in Sc530 was also increased to 10.8 and was 1.80-fold higher than that of pRS425RK in Sc534 (Figure 3A). During the long-term cultivation in non-selective medium, the pE2μ derivate plasmid showed higher stability in PCN and less cell-to-cell variation comparing with pC2μ derivate plasmid. The CV% of Sc530 was increased by 37% from 132.32 (0 generation) to 182.59 (90 generation) (Figure 3C) and the average PCN of p425RT decreased by 38.2% to 6.67 (Figure 3A); While CV % of Sc594 was only increased by 15.90% from 94.95 to 110.05 (90 generation) and lower than that of Sc530 in any generations (see Figure 3B). The average PCN of pE2μRT only decreased by 8.74% to 16.7 after cultivation of 90 generation in non-selective medium (see Figure 3A), the average expression level of the RFP in Sc594 was obviously higher than that of Sc530 (Supplementary Figure 4B). The stability of the expression for the target gene using optimized pE2μ multi-copy system was proved to undergo very long-term cultivation. The strategy of introduction of *TPI1* into plasmid to substitute the native *TPI1* was proved to optimize both pC2μ derivate plasmid and pE2μ derivate plasmid. And the resulting plasmid pE2μRT showed higher PCN and less cell-to-cell variations. Therefore, pE2μRT was thought to be better choice for overexpression of target genes in high copy.

### Application pE2μ Multi-Copy System for DHAA Production

**FIGURE 4:**
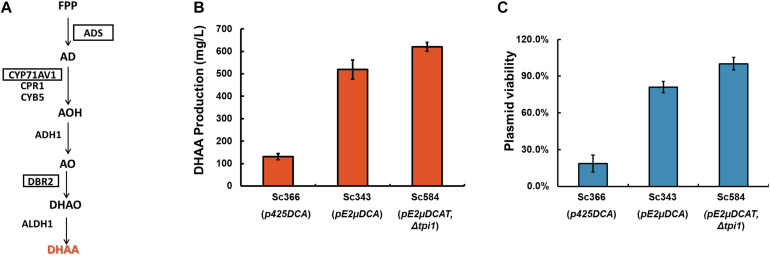
Comparation of the effects of pC2μ and pE2μ multi-copy system on DHAA production. **(A)** The biosynthesis pathway of DHAA. FPP, Farnesyl pyrophosphate; AD, amorpha-4,11-diene; AOH, artemisinic alcohol; AO, artemisinic aldehyde; DHAO, dihydroartemisinic aldehyde; DHAA, dihydroartemisinic acid; AA, artemisinic acid; *ADS*, amorphadiene synthase; *CYP71AV1*, amorphadiene P450 oxidase; *CPR1*, P450 cytochrome reductase from *A. annua*; CYB5, cytochrome b5 from *A. annua*; *ADH1*, artemisinic alcohol dehydrogenase of *A. annua*; *DBR2*, artemisinic aldehyde Δ11(13) reductase of *A. annua*; *ALDH1*, artemisinic aldehyde dehydrogenase of *A. annua. ADS*, *CYP71AV1*, *DBR2* (highlighted in the frame) were genes overexpressed by plasmid systems. **(B)** The DHAA production of strain Sc366, Sc343 and Sc584 in YPD medium. **(C)** The plasmid viability of strains Sc366, Sc343 and Sc584 which were measured at the end of the fermentation in YPD medium.

To demonstrate pE2μ could be applied for optimization of metabolic pathway, dihydroartemisinic acid (DHAA) biosynthesis was chosen as an example. DHAA is the precursor of the anti-malaria drug artemisinin (Paddon and Keasling, 2014). The biosynthesis pathways of DHAA (see Figure 4A) starts from farnesyl pyrophosphate. In this pathway, *ADS*, *CYP71AV1*, and *DBR2* are the key genes for biosynthesis of DHAA and are thought to be overexpressed in high level to increase the production of DHAA. Several studies about the biosynthesis of DHAA in *S. cerevisiae* had been reported (Yansheng et al., 2008; Chen et al., 2017; Zeng et al., 2020). All of these works facilitated pC2μ based plasmids such as pESC-LEU, pRS425, pYES260 to overexpress the key genes of the biosynthesis pathway. Therefore, pE2μ multi-copy system was thought to be helpful for further enhancing the biosynthesis of DHAA by optimization of the plasmid.

In this work, Sc366 was used as the control strain. Sc366 harbored plasmid p425DCA which was constructed based on pRS425 and contained three key genes *ADS*, *CYP71AV1*, and *DBR2*. Strain Sc343 harbored pE2μ plasmid pE2μDCA which contained *ADS*, *CYP71AV1*, and *DBR2*. And Sc584 was modified from Sc343 by introducing the *TPI1* to pE2μDCA (resulting plasmid was plasmid pE2μDCAT) to substitute the native chromosome *TPI1*. In non-selective rich medium (YPD medium), nearly 82% cells of Sc366 lost their plasmid during the fermentation (see Figure 4C), the production of DHAA was only 131.0 mg/L (Figure 4B); For strain Sc343 which harbored plasmid pE2μDCA, only 29.8% of cells lost their plasmid, and the production achieved to 519.2 mg/L and was 3.96-fold higher than that of strain harboring pC2μ plasmid (Figures 4B,C); After optimization with substitution the location of essential *TPI1*, the resulting strain Sc584 didn’t lose the plasmid, and got higher production to 620.9 mg/L. The average copy number of Sc366, Sc343, and Sc584 was 3.33, 6.57, and 9.32, respectively (Supplementary Figure 5). The relationship of mRNA levels of the key genes for different strain was consist with the result of average copy number (Supplementary Figure 6). Comparing with Sc594, the PCN of Sc584 was decreased by about 44.5%. The plasmid stability might be influenced by the overexpression of CYP71AV1 and CPR1, or the rapid accumulation of AA (artemisinic acid) and DHAA. It was consistent with the result of the work of Amyris (Paddon et al., 2013) which found the effect on the plasmid by overexpression of CYP71AV1 or accumulation of product.

The medium with selective pressure for yeast was auxotrophic synthetic medium or the rich medium. However, the non-selective pressure rich medium such as YPD or YPG would help strain to produce more production even the plasmid was not stable (Ro et al., 2008) during the large scale and long-time fermentation (Wang et al., 2017). In rich medium, approximately half of the proteome mass saved by amino acid could be redirected to protein engaged in translation (Bjrkeroth et al., 2020). It could promote cell growth and might increase the expression of the target gene. Therefore, the optimized pE2μ multi-copy system platform was suitable for *S. cerevisiae* to overexpress target gene in non-selective rich medium with higher PCN and has the patent for optimization of biosynthetic pathway and maximize the biochemical production.

In this work, we developed the toolbox for editing the 2μ plasmid to maintain and increase the copy number of the target gene for all *S. cerevisiae* with or without wild type pE2μ (see Supplementary Figure 7). For strain without pE2μ, the plasmid pE2μRAF1 or pE2μREP2 could be used as the backbone directly. The target DNA elements could be inserted into these plasmids *in vitro* or *in vivo*. For strains with wild type pE2μ, the CRISPR/Cas9 plasmid could be used for editing the wild type pE2μ plasmid. And the donor plasmid pDonorRAF1 or pDonorREP2 could be used as the backbone to harbor the target DNA. The example of DHAA production in our work proved that this method could successfully increase the copy number of the target gene. The strain Sc594 was constructed as a convenient chassis for overexpression of the target gene efficiently (Supplementary Figure 7). The pE2μRT could also undergo a serial of editing steps to insert the DNA element of interest. During these steps, extra selective marker is not necessary to insert into the plasmid, and the whole editing process of pE2μ is same to the process for editing the genome of chromosome which had been widely used for optimization of *S. cerevisiae*. And higher copy number of the inserted genes could be easily obtained.

Considering the very low loss frequency of wild type pE2μ, the performance of the loss frequency for pE2μRAF1 and pE2μREP2 was unexpected. Although introduction of essential gene *TPI1* to substitute the original *TPI1* on chromosome could eliminate the plasmid-free cells during the long-term cultivation, the reason for the increasing loss of the plasmid leaded by insertion of foreign DNA into wild type pE2μ plasmid was still needed to be studied. The future study might focus on the regulation of the gene or unknown transcript units of pE2μ plasmid and the stability and the copy number of the pE2μ might be further increased.

## Conclusion

In this work, the endogenous 2μ plasmid had been successfully and perfectly edited by CRISPR/Cas9 system and the donor plasmid. The foreign gene could be inserted to 2μ plasmid in higher copy number for overexpression. The resulting plasmid pE2μ had higher PCN and higher stability than the conventional 2μ based plasmid. In single cell level, the distribution of pE2μ in each cell was more balanced than pC2μ plasmid. Taking these advantages, pE2μ was applied for the heterologous biosynthesis of DHAA, and increased the production titer by 4.73-fold higher than that of control. This study showed the potential of pE2μ for optimization of metabolic pathway by stable and efficiently overexpressing a serial of genes.

## Data Availability Statement

The original contributions presented in the study are included in the article/Supplementary Material, further inquiries can be directed to the corresponding author.

## Author Contributions

B-XZ and YW conceived the study as well as participated in strain construction and carried out the molecular genetic studies. B-XZ and W-HX participated in fermentation. Y-JY, M-DY, Y-ZL, and W-HX participated in design and coordination of the study as well as helped to draft the manuscript. YW supervised the whole research and revised the manuscript. All the authors read and approved the final manuscript.

## Conflict of Interest

The authors declare that the research was conducted in the absence of any commercial or financial relationships that could be construed as a potential conflict of interest.

## References

[B1] Bao Z. Xiao H. Liang J. Zhang L. Xiong X. Sun N. (2014). A Homology Integrated CRISPR-Cas (HI-CRISPR) system for one-step multi-gene disruptions in *Saccharomyces cerevisiae*. *Acs Synthetic Biol*. 4 585–594. 10.1021/sb500255k 25207793

[B2] Bjrkeroth J. Campbell K. Malina C. Yu R. Nielsen J. (2020). Proteome reallocation from amino acid biosynthesis to ribosomes enables yeast to grow faster in rich media. *Proc. Natl. Acad. Sci. U.S.A.* 117 21804–21812. 10.1073/pnas.1921890117 32817546 PMC7474676

[B3] Chai F. Wang Y. Mei X. Yao M. Chen Y. Liu H. (2017). Heterologous biosynthesis and manipulation of crocetin in *Saccharomyces cerevisiae*. *Microb. Cell Fact.* 16:54. 10.1186/s12934-017-0665-1 PMC5371240 28356104

[B4] Chen R. (2012). Bacterial expression systems for recombinant protein production: E. coli and beyond. *Biotechnol. Adv.* 30 1102–1107. 10.1016/j.biotechadv.2011.09.013 21968145

[B5] Chen X. Zhang C. Too H. P. (2017). Multienzyme biosynthesis of dihydroartemisinic acid. *Molecules* 22:1422. 10.3390/molecules22091422 28846664 PMC6151439

[B6] Christianson T. W. Sikorski R. S. Dante M. Shero J. H. Hieter P. (1992). Multifunctional yeast high-copy-number shuttle vectors. *Gene* 110 119–122. 10.1016/0378-1119(92)90454-w 1544568

[B7] Easmin F. Hassan N. Sasano Y. Ekino K. Taguchi H. Harashima S. (2019). gRNA-transient expression system for simplified gRNA delivery in CRISPR/Cas9 genome editing. *J. Biosci. Bioeng*. 128 373–378. 10.1016/j.jbiosc.2019.02.009 31010727

[B8] Entian K. D. Ktter P. (2007). 25 yeast genetic strain and plasmid collections. *Methods Microbiol.* 36 629–666. 10.1016/s0580-9517(06)36025-4

[B9] Futcher A. B. (1986). Copy number amplification of the 2 micron circle plasmid of *Saccharomyces cerevisiae*. *J. Theor. Biol.* 119 197–204. 10.1016/s0022-5193(86)80074-1 3525993

[B10] Gao Y. Zhao Y. (2013). Self- processing of ribozyme- flanked RNAs into guide RNAs in vitro and in vivo for CRISPR- mediated genome editing. *J. Integr. Plant Biol.* 56 343–349. 10.1111/jipb.12152 24373158

[B11] Generoso W. C. Gottardi M. Oreb M. Boles E. (2016). Simplified CRISPR-Cas genome editing for *Saccharomyces cerevisiae*. *J. Microbiol. Methods* 127 203–205. 10.1016/j.mimet.2016.06.020 27327211

[B12] Giaever G. Chu A. M. Ni L. Connelly C. Riles L. Véronneau S. (2002). Functional profiling of the *Saccharomyces cerevisiae* genome. *Nature* 418 387–391. 10.1038/nature00935 12140549

[B13] Gnügge R. Rudolf F. (2017). *Saccharomyces cerevisiae* Shuttle vectors. *Yeast* 34 205–221. 28072905 10.1002/yea.3228

[B14] Jakounas T. Sonde I. Herrgard M. Harrison S. J. Keasling J. D. (2015). Multiplex metabolic pathway engineering using CRISPR/Cas9 in *Saccharomyces cerevisiae*. *Metab. Eng.* 28 213–222. 10.1016/j.ymben.2015.01.008 25638686

[B15] Kang C. W. Gyu L. H. Yang J. Hyun N. M. Woo S. S. Yeol J. G. (2018). Synthetic auxotrophs for stable and tunable maintenance of plasmid copy number. *Metab. Eng.* 48 121–128. 10.1016/j.ymben.2018.05.020 29864582

[B16] Karim A. S. Curran K. A. Alper H. S. (2013). Characterization of plasmid burden and copy number in *Saccharomyces cerevisiae* for optimization of metabolic engineering applications. *Fems Yeast Res.* 13 107–116. 10.1111/1567-1364.12016 23107142 PMC3546148

[B17] Lee C. Kim J. Shin S. G. Hwang S. (2006). Absolute and relative QPCR quantification of plasmid copy number in *Escherichia coli*. *J. Biotechnol.* 123 273–280. 10.1016/j.jbiotec.2005.11.014 16388869

[B18] Lian J. Bao Z. Hu S. Zhao H. (2018). Engineered CRISPR/Cas9 system for multiplex genome engineering of polyploid industrial yeast strains. *Biotechnol. Bioeng*. 115 1630–1635. 10.1002/bit.26569 29460422

[B19] Lian J. Jin R. Zhao H. (2016). Construction of plasmids with tunable copy numbers in *Saccharomyces cerevisiae* and their applications in pathway optimization and multiplex genome integration. *Biotechnol. Bioeng*. 113 2462–2473. 10.1002/bit.26004 27159405

[B20] McQuaid M. E. Pinder J. B. Arumuggam N. Lacoste J. S. C. Chew J. S. K. Dobso M. J. (2017). The yeast 2-μm plasmid Raf protein contributes to plasmid inheritance by stabilizing the Rep1 and Rep2 partitioning proteins. *Nucleic Acids Res*. 45 10518–10533. 10.1093/nar/gkx703 29048592 PMC5737570

[B21] Mignon C. Sodoyer R. Werle B. (2015). Antibiotic-free selection in biotherapeutics: now and forever. *Pathogens* 4:157. 10.3390/pathogens4020157 25854922 PMC4493468

[B22] Misumi Y. Nishioka S. Fukuda A. Uemura T. Nakamura M. Hoshida H. (2019). YHp as a highly stable, hyper-copy, hyper-expression plasmid constructed using a full 2−μm circle sequence in cir0 strains of *Saccharomyces cerevisiae*. *Yeast* 36 249–257. 10.1002/yea.3371 30537227

[B23] Paddon C. J. Keasling J. D. (2014). Semi-synthetic artemisinin: a model for the use of synthetic biology in pharmaceutical development. *Nat. Rev. Microbiol.* 12:355. 10.1038/nrmicro3240 24686413

[B24] Paddon C. J. Westfall P. J. Pitera D. J. Benjamin K. Fisher K. McPhee D. (2013). High-level semi-synthetic production of the potent antimalarial artemisinin. *Nature* 496 528–532. 10.1038/nature12051 23575629

[B25] Piroon J. Thidathip W. Rui P. Preecha P. Ussery D. W. Jens N. (2018). Complete genomic and transcriptional landscape analysis using third-generation sequencing: a case study of *Saccharomyces cerevisiae* CEN.PK113-7D. *Nucleic Acids Res*. 46:e38. 10.1093/nar/gky014 29346625 PMC5909453

[B26] Rizvi S. M. A. Prajapati H. K. Ghosh S. K. (2017a). The 2 micron plasmid: a selfish genetic element with an optimized survival strategy within Saccharmyces cerevisiae. *Curr. Genet*. 64 25–42. 10.1007/s00294-017-0719-2 28597305

[B27] Rizvi S. M. A. Prajapati H. K. Nag P. Ghosh S. K. (2017b). The 2-μm plasmid encoded protein Raf1 regulates both stability and copy number of the plasmid by blocking the formation of the Rep1–Rep2 repressor complex. *Nucleic Acids Res.* 45 7167–7179. 10.1093/nar/gkx316 28472368 PMC5499539

[B28] Ro D.-K. Ouellet M. Paradise E. M. Burd H. Eng D. Paddon C. J. (2008). Induction of multiple pleiotropic drug resistance genes in yeast engineered to produce an increased level of anti-malarial drug precursor, artemisinic acid. *BMC Biotechnol.* 8:83. 10.1186/1472-6750-8-83 18983675 PMC2588579

[B29] Scott E. W. Baker H. V. (1993). Concerted action of the transcriptional activators REB1, RAP1, and GCR1 in the high-level expression of the glycolytic gene TPI. *Mol. Cell Biol.* 13 543–550. 10.1128/mcb.13.1.543 8417350 PMC358933

[B30] Wang R. Gu X. Yao M. Pan C. Liu H. Xiao W. (2017). Engineering of beta-carotene hydroxylase and ketolase for astaxanthin overproduction in *Saccharomyces cerevisiae*. *Front. Chem. Sci. Eng.* 11:89–99. 10.1007/s11705-017-1628-0

[B31] Xie Z. X. Mitchell L. A. Liu H. M. Li B. Z. Liu D. Agmon N. (2018). Rapid and Efficient CRISPR/Cas9-based mating-type switching of *Saccharomyces cerevisiae*. *G3 (Bethesda)* 8 173–183. 10.1534/g3.117.300347 29150593 PMC5765346

[B32] Yansheng Z. Teoh K. H. Reed D. W. Lies M. Alain G. Olson D. J. H. (2008). The molecular cloning of artemisinic aldehyde Delta11(13) reductase and its role in glandular trichome-dependent biosynthesis of artemisinin in Artemisia annua. *J. Biol. Chem.* 283:21501. 10.1074/jbc.m803090200 18495659

[B33] Zeng B.-X. Yao M.-D. Wang Y. Xiao W.-H. Yuan Y.-J. (2020). Metabolic engineering of *Saccharomyces cerevisiae* for enhanced dihydroartemisinic acid production. *Front. Bioeng. Biotechnol.* 8:152. 10.3389/fbioe.2020.00152 32258005 PMC7090239

