# Supplementary material for: Endogenous 2μ Plasmid Editing for Pathway Engineering in Saccharomyces cerevisiae
Source: Front Microbiol. 2021 Feb 16;12:631462. doi: 10.3389/fmicb.2021.631462 (PMC7921170; doi:10.3389/fmicb.2021.631462)
Supplement: Supplementary file 1 [file Data_Sheet_1.docx]

## Supplementary material

# Endogenous 2μ plasmid editing for pathway engineering in *Saccharomyces cerevisiae.*

Bo-Xuan Zeng^1, 2^, Ming-Dong Yao^1, 2^, Wen-Hai Xiao^1, 2^, Yun-Zi Luo^1,3^, Ying Wang^1, 2^*, Ying-Jin Yuan^1, 2^

^1^ Frontier Science Center for Synthetic Biology and Key Laboratory of Systems Bioengineering (Ministry of Education), School of Chemical Engineering and Technology, Tianjin University, Tianjin 300072, China

^2^ Collaborative Innovation Center of Chemical Science and Engineering (Tianjin), Tianjin University, Tianjin 300072, China

^3^ Department of Gastroenterology, State Key Laboratory of Biotherapy, West China Hospital, Sichuan University, Chengdu, 610041, China.


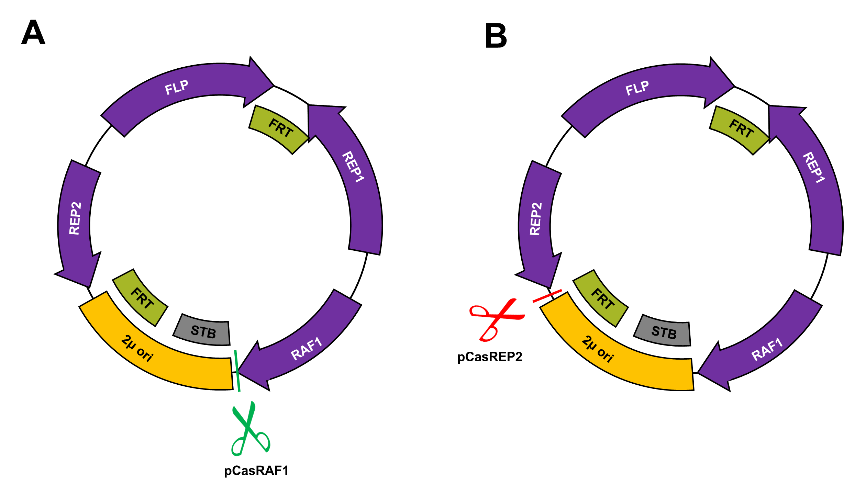


Figure S1. Structure of wild type endogenous 2μ plasmid (pE2μ), two sites were chosen for editing to insert target DNA element.


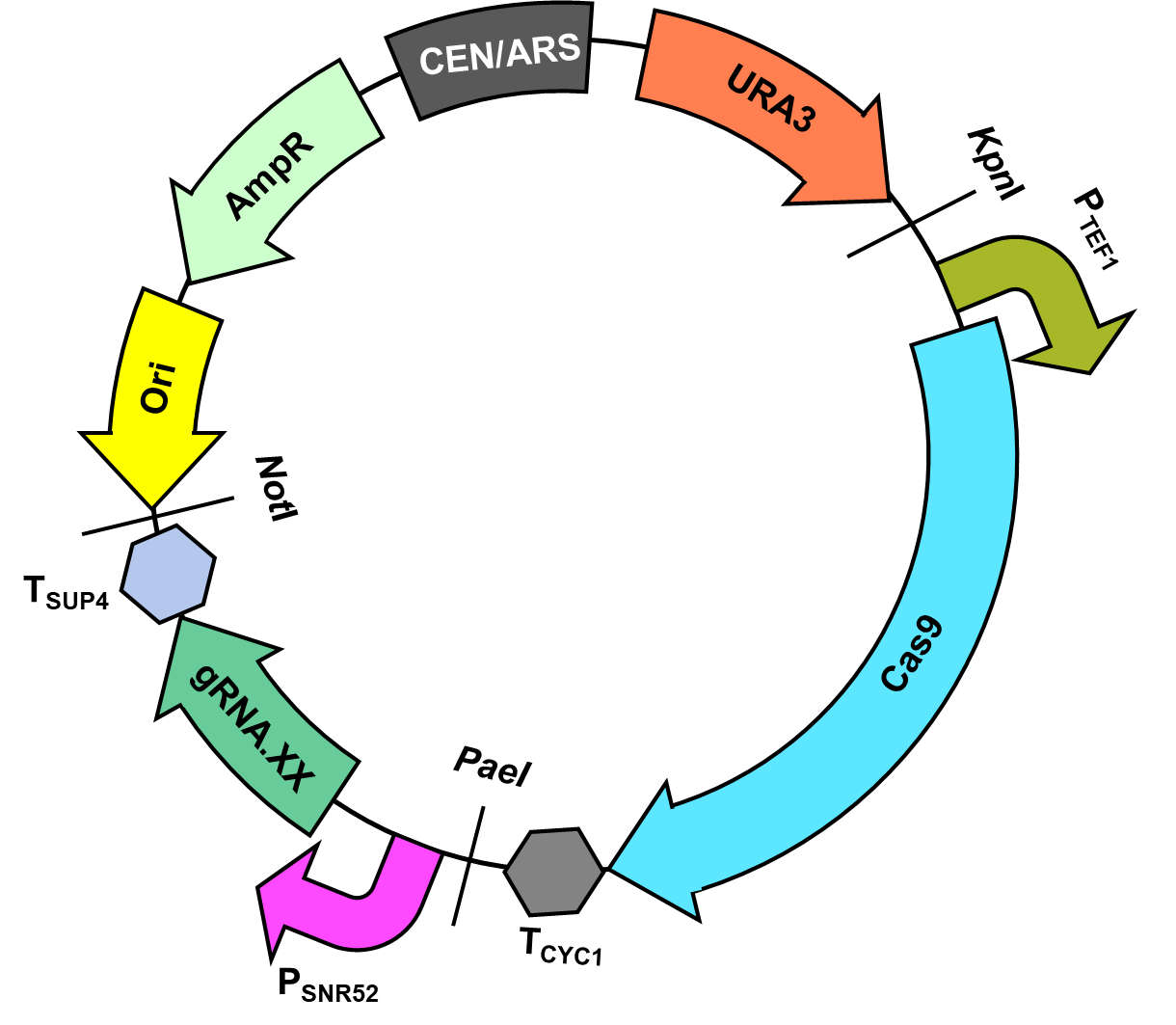


Figure S2 Structure of CRISPR/cas9 plasmid.


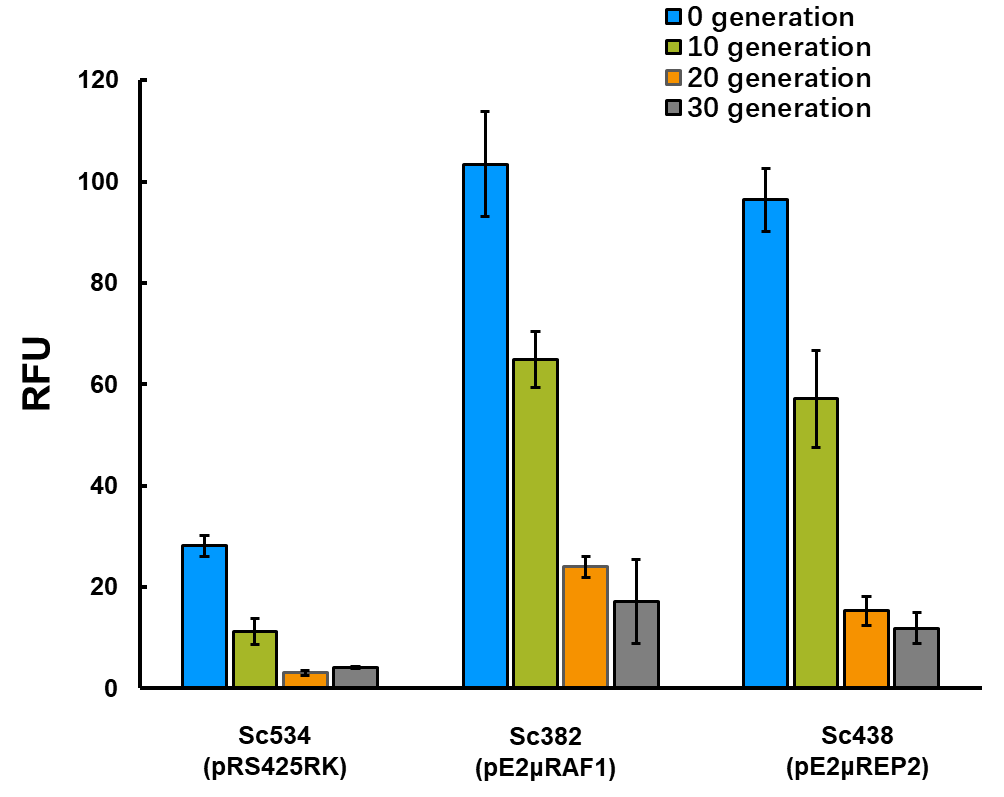


Figure S3. Fluorescence for strain harboring different plasmid in cultivation of 30 generation.


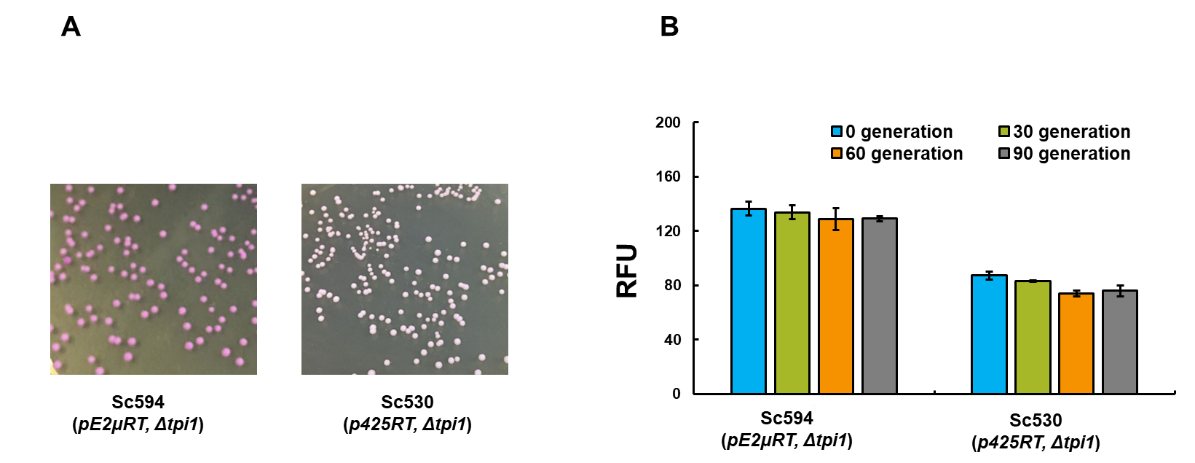


Figure S4. Comparation of Sc594 and Sc530. **(A)** Diluted culture of Sc530 and Sc594 were plated on YPD plate at 90^th^ generation. **(B)** Fluorescence for Sc530 and Sc594 in cultivation of 90 generation.


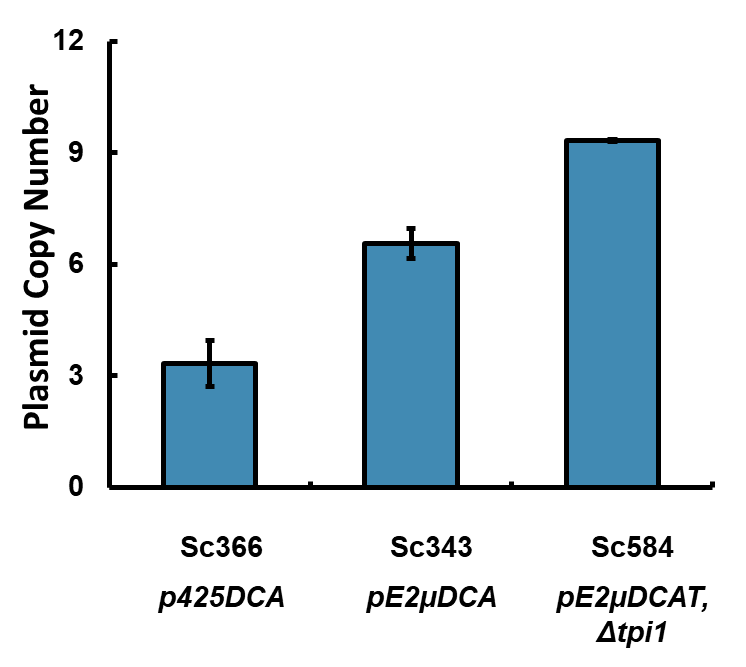


Figure S5. Average PCN of Sc366, Sc343, Sc584 after 120h fermentation.


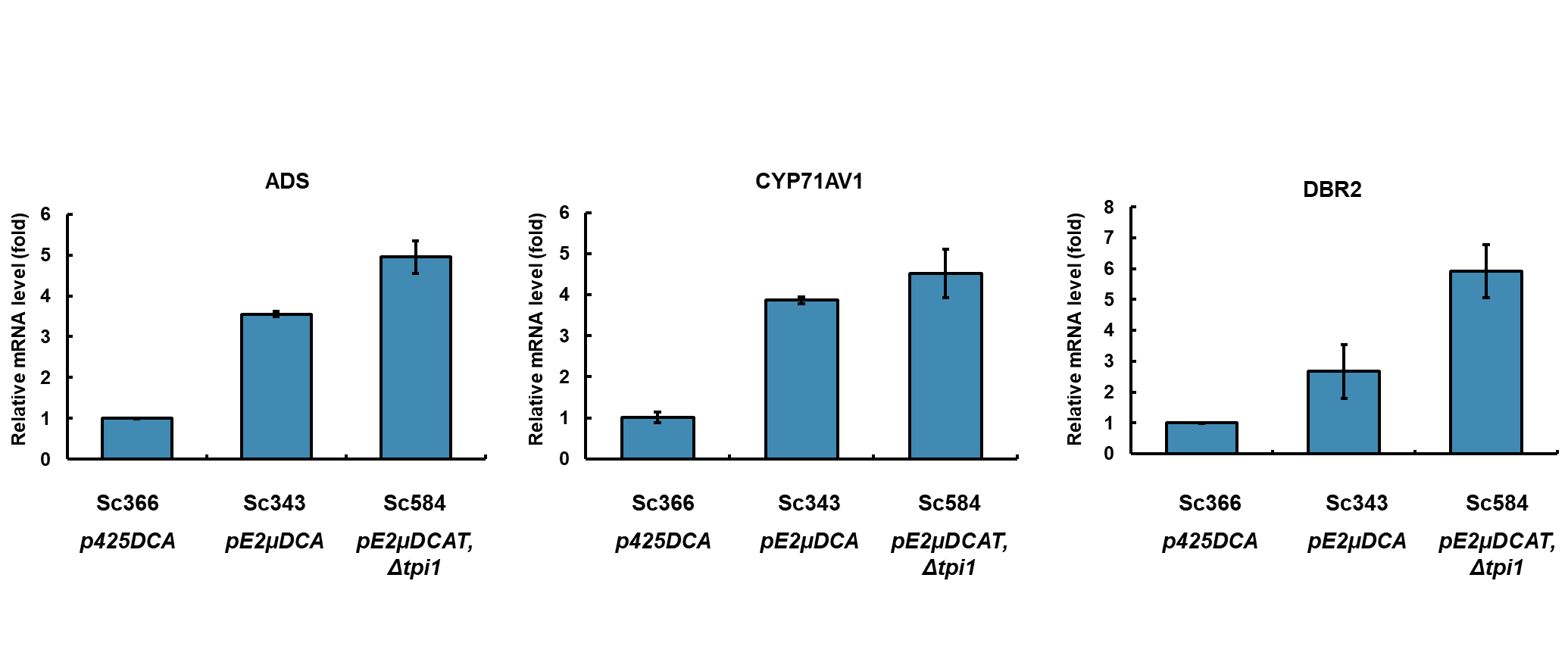


Figure S6. Comparation of relative mRNA level of Sc343 and Sc584 to strain Sc366 for gene ADS, CYP71AV1, DBR2


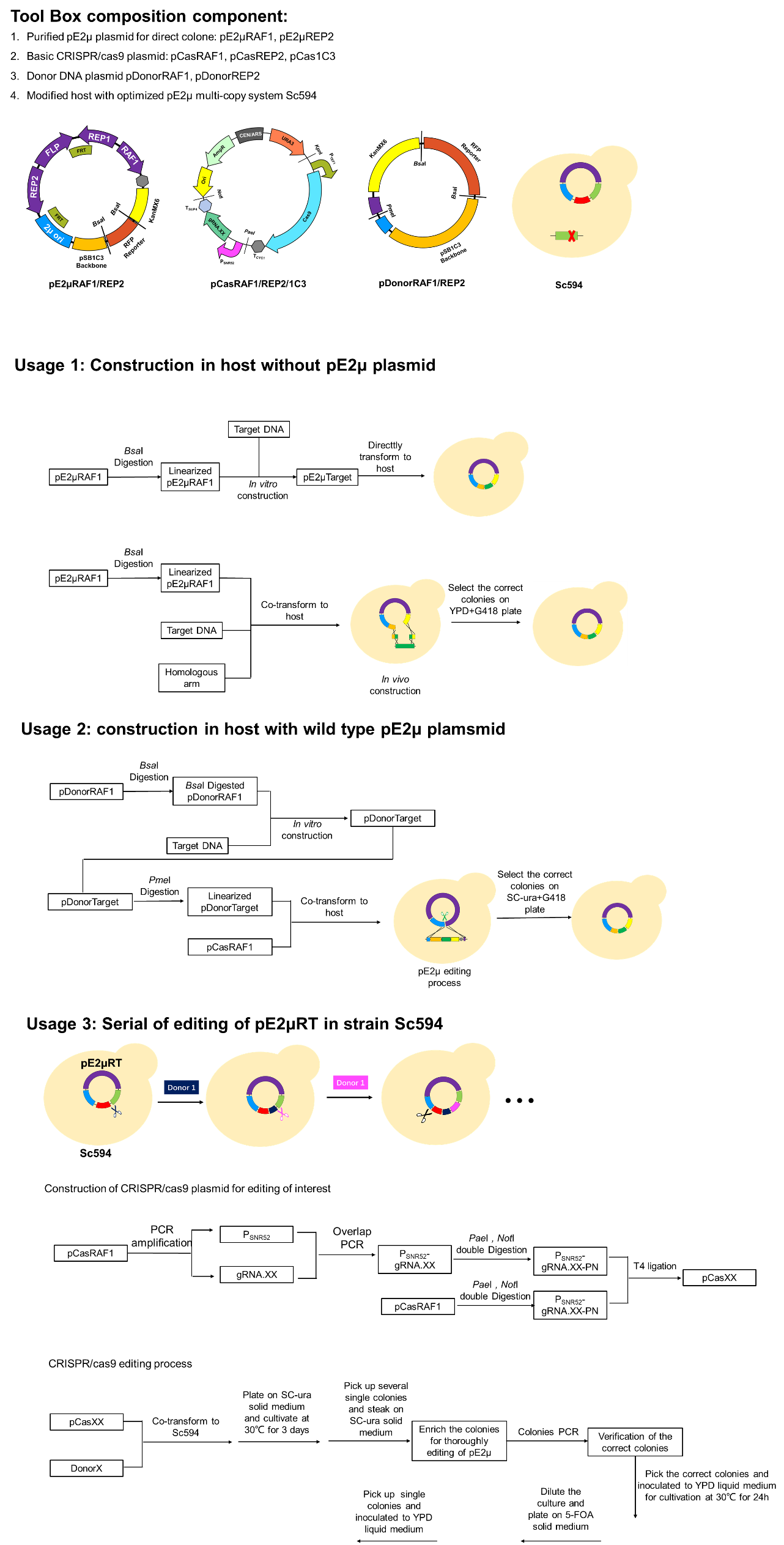


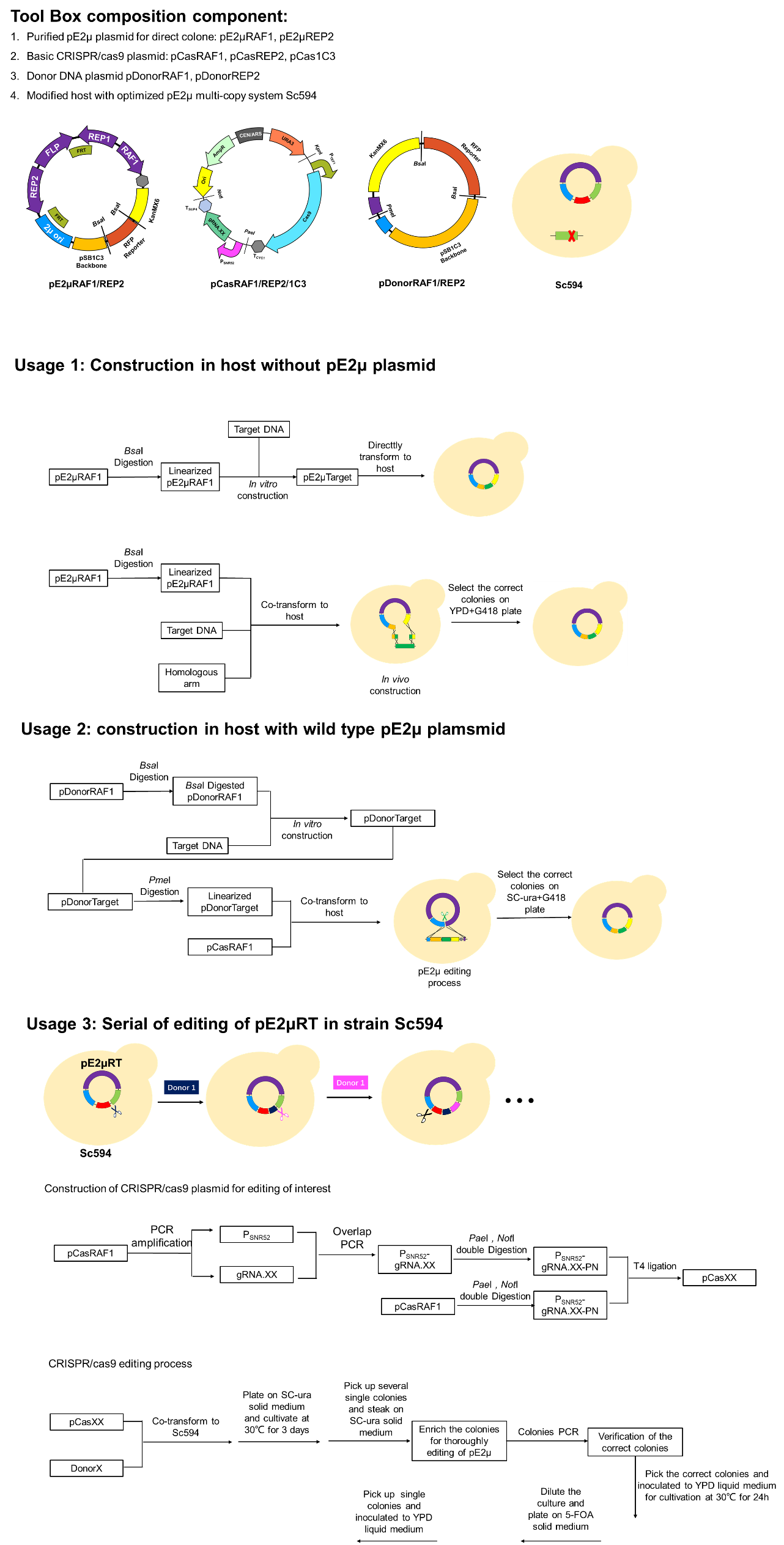


Figure S7. Tool box of the pE2μ multi-copy system for increasing the copy number of the target DNA element in different host.

Table S1. Primer used in this study

| Primer | Sequence |
| --- | --- |
| 18Q2a-pgi1t-R | GTTCGGATGTGATGTGAGAACTGgtagtttagtgtttttcttccagtgcgag |
| 18Q2a-pgi1t-F | aaacgcggatcCAAATCGCTCTTAAATATATACCTAAAGAAC |
| 18Q2a-homodown-F | ttacgcggatccttattatacaggttcaaatatactatctgtttcagggaaaac |
| 18Q2a-homodown-R | atctgtgcttcattttgtaggtttaaactagctagaccgagaaagagactagaaatg |
| 18Q2a-homoup-F | ttctcggtctagctagtttaaacctacaaaatgaagcacagatgcttcgttaacaaag |
| 18Q2a-homoup-R | tttaggactagtcctggagaaactattgcatctattgcatag |
| 18Q4-pTDH3-F | ttaccggaattcggatccgagaccAGTTTATCATTATCAATACTGCCATTTCAAAGAAT |
| 18Q4-adh1t-R | acatacgtctcagtagctcgaggagaccggtagaggtgtggtcaataagag |
| 2μori-test-F | gcgttgcatttttgttctacaaaatg |
| biobrick-R | attaccgcctttgagtgagc |
| 18Q-test-5 | atgccttataaaacagctatagattgc |
| 18Q-test-15 | GATTGCGCCTGAGCGAGACGAAATAC |
| 18Q3a-cas9-F | taacgcggtaccAGCTCATAGCTTCAAAATGTTTCTACTCCTTTTTTACTC |
| 18Q3-cas9-R | gagctcgcatgcCCGCAAATTAAAGCCTTCGAGCGTCCCAAAAC |
| 18Q3-pSNR52-F | taatcgggcatgcgagctcTCTTTGAAAAGATAATGTATGATTATGCTTTC |
| 18Q0b-pSNR52-R | aaacaaatacatacattgtcttccGATCATTTATCTTTCACTGCGGAGAAGTTTCG |
| 18Q0b-gRNA-F | Cggaagacaatgtatgtatttgttttagagctagaaatagcaagttaaaataaggc |
| 18Q3-cyc1t-R | ggataagaatgcggccgcaaagccttcgagcgtcccaaaac |
| 20dTPI-leu2-F1 | CCTTTTCTGGCATCCAGTTTTgattcaagaaatatcttgaccgcag |
| 20dTPI1-leu2-F2 | CAGCTTCCTCTATTGATGTTACACCTGGACACCCCTTTTCTGGCATCCAGTTTTga |
| 20TPI1-leu2-F3 | TTGGTGGAAGATTACCCGTTCTAAGACTTTTCAGCTTCCTCTATTGATGTTACACC |
| 20dTPI1-leu2-R1 | AAAAAGCGCCTTGCTTTTTGTTtgcaccatatcgactacgtcgtaag |
| 20dTPI1-leu2-R2 | tTCAATTGTTAAATGCTTTTCTTCTTTTTATTAGAAAAAGCGCCTTGCTTTTTGTTtg |
| 20dTPI1-leu2-R3 | GTTGATATAGAGGTGTTCAATTGTTAAATGCTTTTCTTCTTTTTATTAGAAAAAGC |
| 20QRcT-F1 | acgtgcccgatcaactcgagtgccacctATCTTCAGTGGCATGTGAGATTCTCC |
| 20QRcT-F2 | cattatggtgaaagttggaacctcttacgtgcccgatcaactcgagtgcc |
| 20QRcT-F3 | caaaaaatacgcccggtagtgatcttatttcattatggtgaaagttggaacctc |
| 20QRcT-R1 | ttaatgtcatgataataatggtttcttGTAAATCTACCGTCCCTTACAAGAAC |
| 20QRcT-R2 | gatacgcctatttttataggttaatgtcatgataataatggtttcttGTAAATCtac |
| 20QRcF-R3 | ttttatctgaaattctgcctcgtgatacgcctatttttataggttaatgtcatg |
| 18Q0d-gRNA-F | GATCgaaaatcacgtaatacttctgttttagagctagaaatagcaagttaaaataaggc |
| 18Q0d-pSNR52-R | gctctaaaacagaagtattacgtgattttcGATCATTTATCTTTCACTGCGGAGAAG |
| 18Q4f0-D-R | CAGATCTCTAGACCATTTGACACTTGATTTGACACTTCTTTTTTTTTTTATTTATG |
| 18Q4f0-D-F | TCAATAGGATCCGTTTAAACGGAAGAAGATGTTATGAAGCTCG |
| 18Q4f0b-2μ-F | GTTTAAACGGATCCCGGATGAAAGGTAGTCTAGTACCTCCTGTG |
| 18Q4f0b-2μ-R | TTATAGCGGCCGCACTAGTTATGATCCAATATCAAAGGAAATGATAGCA |
| qALG9-F | CCAATTGTTTAATCCGGGCTG |
| qALG9-R | CAGTGGACAGATAGCGTAGAG |
| qRFP-F | GTTCATATGGAAGGTTCAGTTAATGGTC |
| qRFP-R | CAAGCAAATGGTAATGGACCACCT |
| qADS-F | GCAATCTTTGGCTAACGACGTTG |
| qADS-R | CAAAGCTGGGTTAGTAGAGAAAGCG |
| qCYP71AV1-F | AATTGCCACTATACTATCCCGTGC |
| qCYP71AV1-R | AGTGATGCAAGAACTTTTTAGATGGG |
| qDBR2-F | TGTGGTGGTTACACCAGAGAATTGG |
| qDBR2-R | GTGGGTGTAGAAGGTAGCTCTGTC |
| 18Q0l-gRNA-F | TCataatggtttcttagacgtcgttttagagctagaaatagcaagttaaaataagg |
| 18Q0l-pSNR52-R | gctctaaaacgacgtctaagaaaccattatGATCATTTATCTTTCACTGCGGAGAAG |
